# Seasonal PrEP for partners of migrant miners in southern Mozambique: a highly focused PrEP intervention

**DOI:** 10.7448/IAS.18.4.19946

**Published:** 2015-07-20

**Authors:** Ide Cremin, Fernando Morales, Britta L Jewell, Kevin R O'Reilly, Timothy B Hallett

**Affiliations:** 1 Department of Infectious Disease Epidemiology, Imperial College London, London, United Kingdom; 2 ICAP Columbia University, New York, NY, USA; 3 World Health Organization, Geneva, Switzerland

**Keywords:** HIV, pre-exposure prophylaxis, ARV-based prevention, cost-effectiveness, mathematical models

## Abstract

**Introduction:**

To be used most effectively, pre-exposure prophylaxis (PrEP) should be prioritized to those at high risk of acquisition and would ideally be aligned with time periods of increased exposure. Identifying such time periods is not always straightforward, however. Gaza Province in southern Mozambique is characterized by high levels of HIV transmission and circular labour migration to mines in South Africa. A strong seasonal pattern in births is observable, reflecting an increase in conception in December. Given the potential for increased HIV transmission between miners returning in December and their partners in Gaza Province, PrEP use by the latter would be a useful means of HIV prevention, especially for couples who wish to conceive.

**Methods:**

A mathematical model was used to represent population-level adult heterosexual HIV transmission in Gaza Province. Increased HIV acquisition among partners of miners in December, coinciding with the miners’ return from South Africa, is represented. In addition to a PrEP intervention, the scale-up of treatment and recent scale-up of male circumcision that have occurred in Gaza are represented.

**Results:**

Providing time-limited PrEP to the partners of migrant miners, as opposed to providing PrEP all year, would improve the cost per infection averted by 7.5-fold. For the cost per infection averted to be below US$3000, at least 85% of PrEP users would need to be good adherers and PrEP would need to be cheaper than US$115 per person per year. Uncertainty regarding incidence of HIV transmission among partners of miners each year in December has a strong influence on estimates of cost per infection averted.

**Conclusions:**

Providing time-limited PrEP to partners of migrant miners in Gaza Province during periods of increased exposure would be a novel strategy for providing PrEP. This strategy would allow for a better prioritized intervention, with the potential to improve the efficiency of a PrEP intervention considerably, as well as providing important reproductive health benefits.

## Introduction

Pre-exposure prophylaxis (PrEP) is a highly effective prevention method, if adhered to [1]. However, PrEP is also expensive to provide. Thus, effective prioritization to those at highest risk of HIV acquisition is essential. A key policy question regarding PrEP is how PrEP should be prioritized and who would be eligible. Much discussion has focused on prioritizing PrEP for key populations such as men who have sex with men, people who inject drugs, sex workers and sero-discordant couples [2, 3], by age [4], and prioritizing within locations with the highest levels of transmission [5, 6]. In addition, PrEP use for an individual would be a short-term method of prevention and therefore should ideally be aligned with specific time periods of high or increased risk of acquisition. However, identifying specific high-risk time periods may not be straightforward for many population groups. Here, we provide a case study of a setting in which a PrEP intervention could potentially be aligned with time periods of increased risk.

Mozambique's HIV epidemic is characterized by strong geographic variation, with the highest levels of prevalence observed in the southern provinces. Population movement, in the form of population displacement, commercial corridors and migrant labour, is thought to have played a considerable role in shaping the epidemiological context and in producing distinct regional epidemics within Mozambique [7]. Since the gold mining industry began in South Africa in the late nineteenth century, there have been high levels of cross-border circular labour migration from the southern provinces of Mozambique (Maputo, Gaza and Inhambane) to mines in South Africa. The earliest Mozambican prevalence data are from the city of Maputo, where HIV prevalence was low and stable until the early 1990s, followed by a rapid increase with prevalence doubling approximately every two years from 1994 onwards [7].

Gaza Province has the highest levels of HIV prevalence in Mozambique. A 2009 AIDS Indicator Survey estimated prevalence to be 30 and 17% among women and men in Gaza Province, respectively [8]. Approximately 40% of Mozambican miners working in South Africa originate from Gaza Province. Within Gaza, the districts of Xai Xai and Chókwè have strong historical ties to mine migration to South Africa, with approximately half of migrant miners from Gaza originating from these two districts [9]. These men typically spend many years working in the South African mines and return to their homes and families in Gaza periodically [9]. A recent survey of men migrating from southern Mozambique to work in South African mines found that, among men from Gaza, almost all have a spouse or long-term partner in Gaza [9]; in addition, casual and commercial sex at the mines is frequent, with 37% of miners reporting at least one “occasional” partner (not a wife or girlfriend) in the last year and 6.6% reporting more than one paid sexual partner in the previous year.

**Figure 1:**
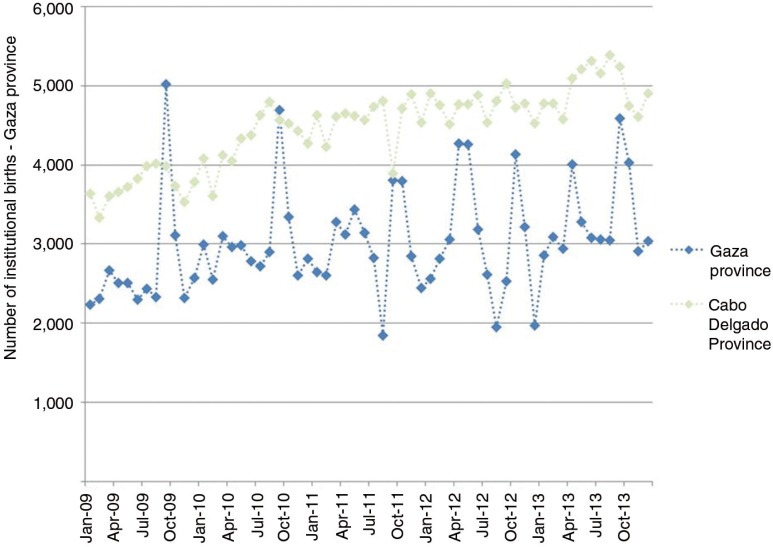
The reported number of institutional births each month in Gaza Province and Cabo Delgado Province, Mozambique.

A seasonal pattern of births is observed in Gaza Province, a high-fertility setting, whereby there is a considerable rise in births each September (Figure 1). Increased frequency of conception during December is hypothesized to be related to labour migrants, including miners, returning from South Africa over the Christmas period. In Mozambique, this seasonal trend in institutional births is specific to the southern region where there is a large volume of labour migration to South Africa; it is not observed in northern provinces, where labour migration is infrequent. Given a seasonal trend of an increase in unprotected sex in December, it can be hypothesized that HIV transmission would also increase during this time period. Providing PrEP to partners of migrant miners to coincide with the miners’ return to Gaza for Christmas would allow a well-defined location, population and time for a PrEP intervention. Furthermore, it would provide reproductive health benefits by allowing these women to conceive while also receiving protection from HIV transmission. The aim of this paper is to estimate the prevention impact and the cost-effectiveness of providing time-limited PrEP to partners of migrant miners in Gaza, Mozambique.

## Methods

Following previous work, we used a deterministic population-level model of heterosexual HIV transmission [10]. The model is stratified according to the natural history of HIV infection, sex, male circumcision, three behavioural risk groups and PrEP use. The model is parameterized for Gaza Province and a full model definition is provided in the technical appendix.

Migrant miners and their partners in Gaza are each represented as a behavioural risk group. The proportion of men who are miners is based on the number of men from Gaza who migrate to work in the mines in South Africa, approximately 15,000 men in 2011. This represents 5% of all men in Gaza, and their partners are assumed to represent an equivalent fraction of all women in Gaza. Time-limited transmission is represented such that all transmission between miners and their partners in Gaza is restricted to December each year. Either the miners or their partners can be infected by another partner at any other time throughout the year.

Infection among the miners that occurs in the mining communities in South Africa is represented in the model as a separate incidence term that the miners are subject to for the first 11 months of each year. This incidence is calibrated to produce the observed level of prevalence among migrant miners from Gaza [11, 12].

Self-reported behavioural data indicate that condom use between migrant miners and their regular partners in Mozambique is very infrequent, with the majority (80%) of miners reporting that they had not used a condom at all with their regular partner in Mozambique at any point in the previous year [9, 12]. For simplicity, it is assumed that all sex between miners and their partners in Gaza is condomless and sex acts are frequent throughout December.

The large expansion of antiretroviral therapy (ART) in Gaza Province since 2005 is represented in the model (Supplementary Figure 3). In 2013, there were approximately 43,000 individuals receiving ART, with a larger number of women receiving ART than men. The scale-up of ART as represented in the model corresponds to coverage among all infected individuals, reaching 40% by 2017 and remaining stable at that level beyond 2017. A dropout rate of 5 per 100 person-years (PY) is assumed. ART is assumed to reduce infectiousness by 91%, as reported in a systematic review and meta-analysis of HIV-1 infectiousness per heterosexual partnership based on prospective studies of sero-discordant couples [13].

The recent scale-up of voluntary medical male circumcision in Gaza Province is also represented. Prior to the scale-up of a male circumcision intervention in 2010 [14], the percentage of men that were circumcised is assumed to be low at approximately 20%, as observed for Gaza Province in the 2003 and 2011 Demographic and Health Surveys [15, 16]. The prevalence of male circumcision is assumed to be the same across all adult men in Gaza Province, including the miners. Data regarding the number of men newly circumcised in Gaza Province was used to approximate how the overall prevalence of male circumcision in Gaza would be expected to increase up to 2013. Male circumcision is assumed to reduce the risk of acquisition by 60% [17–19] and have no effect on onward transmission once infected.

**Figure 2:**
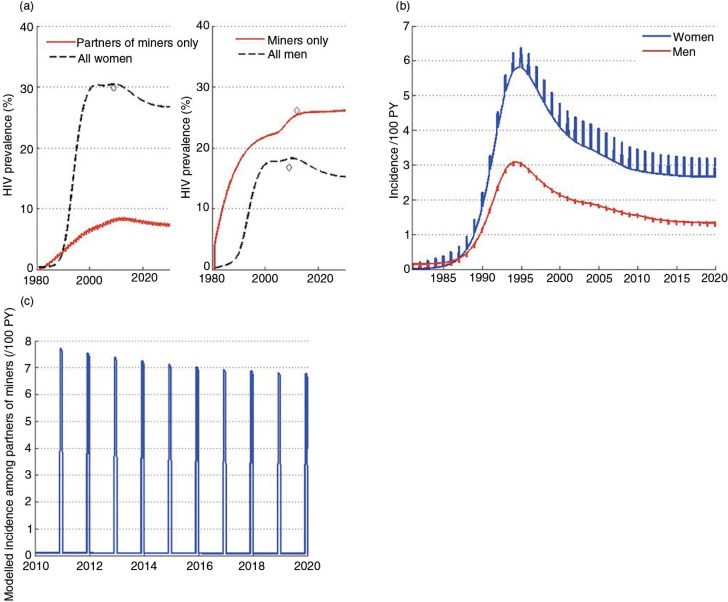
Modelled prevalence and incidence. Model calibration to prevalence (a); modelled incidence among men and women (b); modelled incidence among partners of miners (c).

The model is calibrated to sex-specific prevalence estimates for Gaza Province from a 2009 AIDS Indicator Survey (Figure 2). A prevalence estimate for miners, from Gaza specifically, was obtained from a recent survey among Mozambican men working in South African mines [11] and was found to be 26% [11]. A prevalence estimate among partners of miners was not available to calibrate the model – an important limitation. Modelled prevalence among partners of miners is 2.6 times lower than prevalence among the miners, due to an assumed lack of exposure throughout the rest of the year. In settings within South Africa, a similarly high differential in prevalence between men migrating long distances to work in the mines and their rural partners has been observed. From the migrant-sending community of Hlabisa in rural KwaZulu-Natal, men who migrated to work in the gold mines in Carletonville, a destination 700 km away, were 3.2 times more likely to be infected as their rural partners, unlike men who migrated from Hlabisa to work in nearby mines, who had similar prevalence to their rural partners [20]. If the partners of miners in Gaza have a true prevalence significantly higher than the modelled estimate, or their patterns of risk behaviour vary substantially, the projected effect of the intervention could be reduced.

The model includes a representation of a PrEP intervention. The efficacy of PrEP per protected sex act is assumed to be 91% [21], with protection dependent on user adherence. PrEP users are split into two groups of adherers – “poor” (20% of users) or “good” (80% of users) – with a different proportion of their sex acts protected. Only uninfected partners of miners receive PrEP. A fixed percentage of partners receive PrEP for the last six weeks of each year. An additional two weeks of PrEP is provided in order to establish sufficient drug concentration to receive protection for the final four weeks of the year. Analyses are presented for a five-year intervention period (2015 to 2020).

The cost of ART is assumed to be US$294 per person per year, based on recent estimates of total per-patient costs for established adult ART patients on first-line regimens in Mozambique [22]. Given that the cost of providing PrEP in this setting is uncertain, a range is explored, with a maximum of US$300 per person per year, as it is assumed that it would not be substantially more expensive to provide PrEP than to provide ART. ART spending is included in the cost calculations to capture the effect of reduced future ART spending due to infections averted by means of PrEP. The net spending is calculated as the difference between intervention spending (PrEP and ART) and baseline spending (ART only). A health system perspective is used to calculate cost per infection averted. All costs are discounted annually by 3%.

## Results

The model calibration to HIV prevalence data is shown in Figure 2a. The large gender differential in prevalence is captured whereby prevalence among women in 2009 is approximately 30%, and prevalence among men in 2009 is much lower, at approximately 17%. Prevalence among the miners is higher at 26% [11]. Prevalence among women from the early 2000s to 2009 is consistent with ANC sentinel surveillance data from Gaza Province, during which time prevalence increased dramatically and subsequently stabilized.

Modelled incidence is higher for women than men (Figure 2b), and spikes in incidence are due to increased transmission between the miners and their partners each December. Incidence among both men and women is projected to decrease gradually and stabilize in the near future. The representation of time-limited transmission between miners and their partners in Gaza in the model produces a spike in the modelled incidence among partners of the miners each December (Figure 2c).

**Figure 3:**
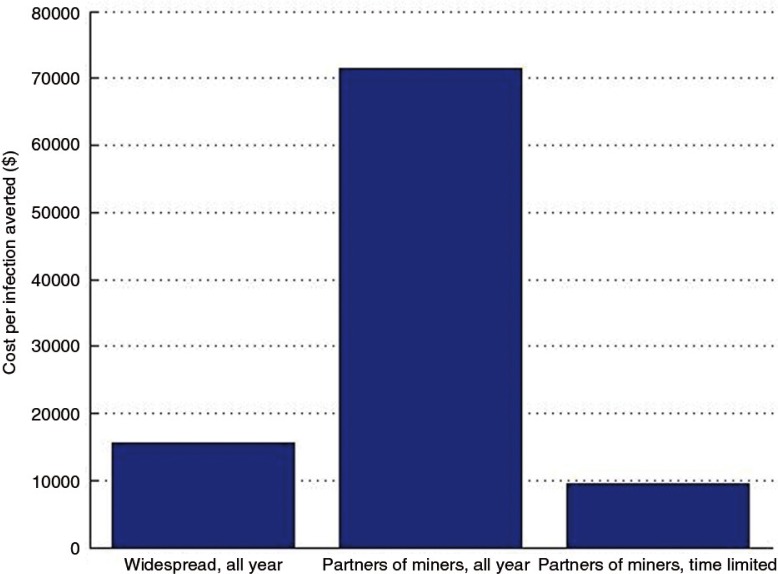
The influence of prioritizing PrEP on the estimated cost per infection averted.

Assuming that PrEP costs US$300 per person per year and that all uninfected women are eligible to receive PrEP, the cost per infection averted is US$15,647 (Figure 3). Providing PrEP specifically to partners of miners increases the cost per infection averted by over fourfold, to US$71,374. This number reflects the relative level of incidence among partners of miners specifically (who have low incidence throughout the year and are assumed to only have elevated incidence for one month each year) as compared to all women in Gaza. However, providing PrEP to partners of the miners for only the last six weeks of the year reduces the cost per infection averted dramatically, to US$9538. Although much less PrEP is being paid for, a similar number of infections are being averted, as the time period of PrEP use corresponds to the time period of increased incidence.

**Figure 4:**
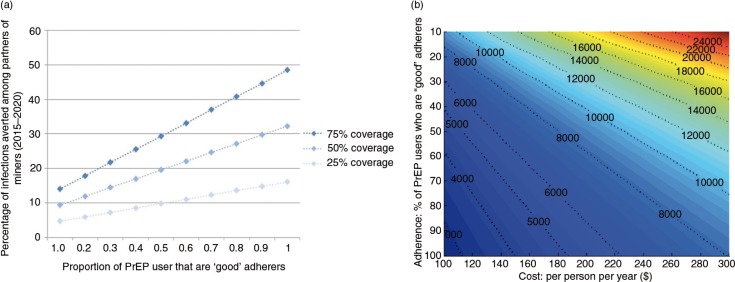
The (a) influence of adherence on impact and (b) influence of adherence and cost on cost per infection averted.

Next, a time-limited PrEP intervention for partners of miners is further explored. Adherence determines impact and poor adherence will degrade impact. The level of adherence is unknowable in advance for a myriad of reasons, and thus a wide range is explored. The relationship between adherence and impact, among partners of the miners specifically, is illustrated for several levels of coverage (Figure 4a).

Assuming no economies of scale, as is assumed here, the cost per infection averted is constant over coverage. The influence of adherence and cost on cost per infection averted (among the adult population of Gaza Province), assuming 50% coverage, is illustrated in Figure 4b. For the cost per infection averted to be below US$3000, at least 85% of PrEP users would need to be good adherers and PrEP would need to be cheaper than US$115 per person per year.

The true level of HIV incidence in December among partners of migrant miners and the proportion of new infections among partners of the miners that originate from the miners, as opposed to external partners, are unknown. Modelled incidence among partners of the miners is approximately 7 per 100 PY in December, based on the default model parameterization. In the model, the majority (87%) of new infections each year are from the miners (when they return in December) and the remaining 13% are acquired from other partners throughout the year. The true incidence in December is important as it determines the scope of impact of the PrEP intervention. Therefore, analyses are carried out whereby the incidence in December is lower.

**Figure 5:**
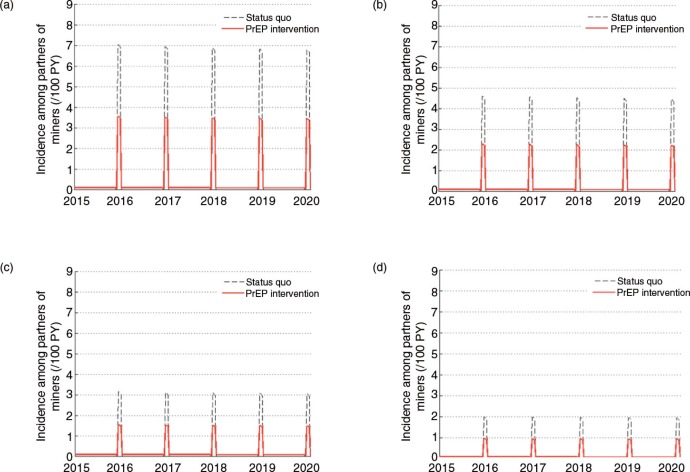
The influence of different assumptions regarding the level of transmission occurring between miners and their partners in December.

The relative impact (as opposed to absolute impact) is similar regardless of the underlying incidence in December (Figure 5, Table 1). As expected, there is an inverse relationship between incidence in the status quo scenario and cost per infection averted. If incidence among partners of miners in December was 4.6 per 100 PY – an incidence rate observed among high-risk women in Chókwè district in Gaza Province [23] – the estimated cost per infection averted would be US$14,868. If incidence among partners of miners in December was much lower at 2 per 100 PY, the estimated cost per infection averted would be as high as US$32,680.

**Table 1:** The percentage of infections averted among partners of miners and the discounted cost per infection averted for different assumptions regarding the level of transmission occurring between miners and their partners in December.

	(a) 7 per 100 PY (default)	(b) 4.6 per 100 PY (as observed among high risk women in Chókwè	(c) 3 per 100 PY	(d) 2 per 100 PY
Percentage of infections averted among partners of miners (2015–2020)	43.5%	42.0%	39.9%	36.0%
Discounted cost per infection averted ($)	$9,998	$14,868	$21,262	$32,680

Overall, the estimated costs per infection averted would be considerably lower if PrEP was provided for four or five weeks, rather than six, given that modelled incidence is very low in the weeks prior to the miners’ return in December.

## Discussion

Providing PrEP to partners of migrant miners in Gaza Province during periods of increased exposure would be a novel strategy for providing PrEP (i.e. time-limited PrEP), allowing for a better prioritized intervention with the potential to improve the efficiency of a PrEP intervention considerably, minimize potential side effects and provide reproductive health benefits. Key uncertainties include risk behaviour and HIV incidence among men while at the mines, risk behaviour of their partners in Gaza while the miners are away and behaviour in December when the miners return.

Partners of migrant miners in southern Mozambique have been identified as a vulnerable population, given very low condom use and the disproportionately high HIV prevalence among migrant miners [9]. Risk perception among these women is high, with three-quarters of partners of migrant miners surveyed reporting they felt they were at high risk of becoming infected [9]. Absence of condom use has been attributed to lack of power to negotiate condom use within marriage [9]. Fertility intentions among these women may be an additional barrier to condom use. A 2006 survey among rural married women in Gaza Province found that approximately 70% of women wanted to have more children. Furthermore, desire for more children was higher among migrants’ wives compared to non-migrants’ wives, 76.1 vs 64.3%, respectively [23]. Thus, PrEP would be a useful prevention option for women who are at increased risk of HIV acquisition and also wish to conceive.

A number of important simplifications are made in the model. All HIV transmission between the miners and their partners in Gaza is assumed to happen in the month of December each year, whereas in reality some miners return to Gaza more frequently. Previous modelling found that, for a given level of risk behaviour among migrants, the frequency of returning home strongly influenced the risk of acquiring HIV among rural partners, particularly in the early stages of the epidemic [20]. However, this increased risk was outweighed by the influence of increases in risk behaviour due to circular labour migration [20]. In addition, we assume that the partners of miners experience very low incidence for the duration of the time when the miners are away. If the partners of miners have more external partners than assumed, providing time-limited PrEP would not protect them from other sources of infection throughout the year, though this risk would not substantially affect the cost per infection averted. Finally, uninfected partners of miners are assumed to all have the same risk of acquiring HIV. In practice, any effort to further prioritize these women with respect to risk of acquisition would be beneficial. The model was not stratified by age, and prioritizing PrEP for younger women could potentially improve the cost-effectiveness of the intervention.

The strength of the rationale for such a prioritized time-limited PrEP intervention depends on the following: the extent to which there is an increase in births in September; the extent to which this increase can be attributed to the miners’ return at Christmas; and the extent to which an increase in conception around this time leads to an increase in HIV transmission. The data presented reflect institutional births only, which account for approximately 60% of all births. Thus, the true pattern of monthly births might be either more or less pronounced if the remaining 40% of births, that is, home births, were also included. It is unknown how much of the increase in births in September can be attributed to a high frequency of unprotected intercourse following the return of migrant workers at Christmas, as opposed to a behavioural trend unrelated to migration. There are other labour migrant populations who migrate seasonally from Gaza to neighbouring high prevalence countries, for example farm workers, who are likely to be contributing to the observed seasonal pattern of births. However, time-limited PrEP for the partners of miners would still be a useful intervention if the September increase is partially attributable to the miners’ return at Christmas. Many of the miners return to Gaza for two weeks at Easter, yet this return does not manifest in the monthly pattern of births. A possible “Easter effect” would be attenuated by the fact that not all miners return at Easter, as it is a much shorter time period and the timing of Easter varies each year. It would be difficult to quantify the increase in unprotected sex that would be required to produce the increase in births observed in September. Furthermore, quantifying the increase in HIV transmission by translating an increase in unprotected sex would have many uncertainties. Additional data would be required to establish and directly quantify an increase in HIV acquisition specifically among partners of miners during December before implementing such a PrEP intervention.

Given a strong rationale for the proposed PrEP intervention, the feasibility and practicalities of such an intervention need to be addressed. Firstly, the health-care system in rural Mozambique is particularly weak, with less than 40% of the population having access to basic services [9], although there have been improvements in recent years. Secondly, basic HIV prevention services need to be strengthened and expanded. Thirdly, changes in recruitment policies will result in a steady decline in the number of Mozambican men working in the mines in South Africa. Despite this changing situation, a PrEP intervention in Gaza would still be worthwhile because it would protect partners of men who are currently mining. Finally, such a tailored PrEP intervention would need to be carefully executed to avoid the miners and their partners feeling singled out and consequently stigmatized. The acceptability of PrEP for this population and willingness to adhere would ideally be assessed in advance of implementing this intervention. Furthermore, one challenge of implementing such an intervention will be developing a strategy for identifying miners and their partners in Gaza.

Dynamic models of infectious disease transmission have many uses, one of which is as a tool to aid planning and evaluation of interventions [24, 25]. Mathematical models provide a framework to collate several sources of data and make projections of epidemiological impact and cost-effectiveness of an intervention under different sets of assumptions. This analysis provides an example of how mathematical modelling can be useful to inform policy-relevant decision making. Challenges include the availability of data for model calibration and parameterization, as well as uncertainty about patterns of sexual behaviour in the population modelled. Other cost-effectiveness analyses of PrEP have predicted the cost per infection averted to range between cost-saving and US$67,000, depending on the intervention strategy and behavioural characteristics of the population [4]. Providing time-limited PrEP to the partners of miners in Mozambique is more cost-effective than many strategies investigated previously, and it has the potential to become even more cost-effective if the price of PrEP is lower than assumed.

The analyses for this population may be broadly relevant for other migrant groups in different settings, for whom a prioritized intervention such as PrEP would be beneficial. The epidemiological context in Gaza Province provides a novel opportunity for time-limited PrEP, allowing HIV prevention and reproductive health benefits. Beyond a clear need for additional HIV prevention in Gaza Province, seasonal HIV exposure and transmission would provide a niche for a well-prioritized PrEP intervention.

## Supplementary Material

Seasonal PrEP for partners of migrant miners in southern Mozambique: a highly focused PrEP intervention
