# Supplementary material for: Seasonal PrEP for partners of migrant miners in southern Mozambique: a highly focused PrEP intervention
Source: J Int AIDS Soc. 2015 Jul 20;18(4Suppl 3):19946. doi: 10.7448/IAS.18.4.19946 (PMC4509898; doi:10.7448/IAS.18.4.19946)
Supplement: Seasonal PrEP for partners of migrant miners in southern Mozambique: a highly focused PrEP intervention [file JIAS-18-19946-S001.pdf]

Supplementary material for:

Seasonal PrEP for partners of migrant miners in Gaza, southern Mozambique - A highly focused PrEP intervention

Ide Cremin, Fernando Morales, Britta L. Jewell, Kevin O'Reilly, Timothy B. Hallett

Model overview

The model is a deterministic compartmental model defined by a set of ordinary differential equations which are solved numerically in Matlab. A time step of one week (0.019) is used. The state variables are denoted by $X_{l,k}^{s,p}$ where:

- s refers to HIV status (s=0 susceptible, s=1 acute infection, s=2 chronic infection with CD4 >350 cells/μL, s=3 chronic infection with CD4 >200 cells/μL but <350 cells/μL, s=4 late infection with CD4 <200 cells/μL, s=5 pre-AIDS with heightened infectiousness, s=6 AIDS, s=7 ART >350 cells per μl and s=8 ART <350 cells per μl);

- p refers to PrEP status (p=1 not eligible for PrEP, p=2 eligible for PrEP, p=3 receiving PrEP and adhering well, p=4 receiving PrEP and adhering poorly);

- l refers to behavioural risk group (l=1 low risk, l=2 intermediate risk, l=3 migrant miner (if male), partner of migrant miner (if female));

- k refers to sex and male circumcision (k=1 females, k=2 uncircumcised males, k=3 circumcised males, k=4 newly circumcised males in the healing period);

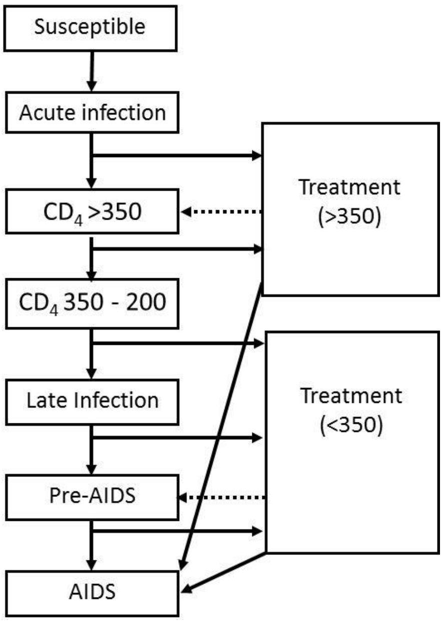

Figure S1. Flow diagram illustrating model representation of the natural history of HIV infection and ART initiation. Dashed arrows indicate treatment drop-out.

Natural history of infection

$$\frac{dX_{l,k}^{0,p}}{dt}=(\mu+\alpha)N\Psi_{(l,k,p)}-(\lambda_{l,k}^{p}+\mu)X_{l,k}^{0,p}+g(k)+h(p)$$

$$\frac{dX_{l,k}^{1,p}}{dt}=\lambda_{l,k}^{p}X_{l,k}^{0,p}-(\sigma_1+\mu)X_{l,k}^{1,p}+g(k)$$

$$\frac{dX_{l,k}^{2,p}}{dt}=(1-\phi_1)\sigma_1X_{l,k}^{1,p}+\xi_E X_{l,k}^{7,p}-(\sigma_2+\mu)X_{l,k}^{2,p}+g(k)$$

$$\frac{dX_{l,k}^{3,p}}{dt}=\left(1-\phi_2\right)\sigma_2X_{l,k}^{2,p}-(\sigma_3+\mu)X_{l,k}^{3,p}+g(k)$$

$$\frac{dX_{l,k}^{4,p}}{dt}=\left(1-\phi_3\right)\sigma_3X_{l,k}^{3,p}-(\sigma_4+\mu)X_{l,k}^{4,p}+g(k)$$

$$\frac{dX_{l,k}^{5,p}}{dt}=\left(1-\phi_4\right)\sigma_4X_{l,k}^{4,p}-(\sigma_5+\mu)X_{l,k}^{5,p}+\xi_L X_{l,k}^{8,p}+g(k)$$

$$\frac{dX_{l,k}^{6,p}}{dt}=\left(1-\phi_5\right)\sigma_5X_{l,k}^{5,p}+\omega_E X_{l,k}^{7,p}+\omega_L X_{l,k}^{8,p}-(\gamma+\mu)X_{l,k}^{6,p}+g(k)$$

$$\frac{dX_{l,k}^{7,p}}{dt}=\phi_1\sigma_1X_{l,k}^{1,p}+\phi_2\sigma_2X_{l,k}^{2,p}-(\omega_E+\xi_E+\mu)X_{l,k}^{7,p}+g(k)$$

$$\frac{dX_{l,k}^{8,p}}{dt}=\phi_3\sigma_3X_{l,k}^{3,p}+\phi_4\sigma_4X_{l,k}^{4,p}+\phi_5\sigma_5X_{l,k}^{5,p}-(\omega_L+\xi_L+\mu)X_{l,k}^{8,p}+g(k)$$

…(1)

For a given infection stage, rates of progression to the next stage are given by the rates σ_s $(s=1,2,3,4,5)$ (Table S1). The late infection stage is defined by the mean time between the state of infection when CD4 count falls below 200 copies/μL and viremic rebound 19 months, on average, prior to death. The pre-AIDS stage is a characterization of the 9 month period of heightened infectiousness before the AIDS stage, which is assumed to be a 10 month period of no transmission risk as per Hollingsworth et al. [1]. A limitation of this model is that AIDS-related mortality (γ) only applies to this final AIDS stage, whereas in reality some infected individuals may die of AIDS-related illnesses at higher CD4 counts.

Parameter	Value	Source
Mean length of time in acute HIV infection phase	2.9 months	As estimated by Hollingsworth et. al. [1]
Mean time from end of acute HIV infection to CD4 count = 350	4.56 years	Based on a mean duration of 4.8 years from sero-conversion to CD4 <350 reported in Wandel et. al. [2]<br>(4.8 – 0.24 (2.9 months) = 4.56)
Mean time from CD4 count = 350 to CD4 count = 200	4.6 years	Based on a mean duration of 9.4 years from sero-conversion to CD4 <200 reported in Wandel et. al. [2]<br>(9.4 - 4.56 – 0.24 = 4.6)
Mean time from CD4 count < 200, before pre-AIDS I	3.62 years	Based on a mean duration of 5.2 years from CD4 <200 to death reported in Wandel et. al. [2]<br>(5.2 years – 1.58 years (19 months) = 3.62)
Duration of time with higher infectiousness due to acute HIV infection	9 months	As estimated by Hollingsworth et. al. [1]
Duration of time with no HIV transmission before death	10 months	As estimated by Hollingsworth et. al. [1]

Table S1: Assumptions regarding natural history of HIV infection, in the absence of competing hazards from non-HIV mortality.

Population structure

The simulated population size is calibrated to match estimates of the adult population of Gaza province. Individuals enter the population at a rate μ +α (where α is the population growth rate (2.3%)). All individuals are assumed to enter the susceptible population. A rate of exit from the population μ (1/35) is applied to each state.

The total number in the population (N) is:

$$N = \sum_{l=1}^{3}\sum_{k=1}^{4}\sum_{p=1}^{4}\begin{pmatrix} X_{l,k}^{0,p} + X_{l,k}^{1,p} + X_{l,k}^{2,p} + X_{l,k}^{3,p} + X_{l,k}^{4,p} \\ + X_{l,k}^{5,p} + X_{l,k}^{6,p} + X_{l,k}^{7,p} + X_{l,k}^{8,p} \end{pmatrix}$$

…(2)

$\Psi_{(l,k,p)}$ is the matrix of population distribution in the year the epidemic starts (t_0) over each l,k,p stratum and it is defined in terms of $\psi_{f\,(l)}$ and $\psi_{m\,(l)}$ which are the proportion of females and males in each behaviorual activity group. The proportion of men from Gaza province who migrate to work

in the South African mines is assumed to be 5.6%, based on the number of miners reported to officially migrate from Gaza province.

The parameter f_{cm} gives the fraction of males that enter adulthood already circumcised. It is assumed that men do not enter the population in the circumcision healing period $(k=4)$.The parameter $f_{\mathrm{Pr}EP}$ gives the fraction of individuals eligible for PrEP (defined separately by sex and risk group). $\Psi_{(l,k,p)}$ is given by:

$$\Psi_{(l,k,p)} = \begin{cases} \tfrac{1}{2}\psi_{f(l)}\,(1\text{-}f_{\mathrm{Pr}EP}) & \text{any } l; k=1;\ \mathrm{p}=1; \\ \tfrac{1}{2}\psi_{f(l)}\ f_{\mathrm{Pr}EP} & \text{any } l; k=1;\ \mathrm{p}=2; \\ \\ \tfrac{1}{2}\psi_{m(l)}(1-f_{cm})(1\text{-}f_{\mathrm{Pr}EP}) & \text{any } l; k=2;\ \mathrm{p}=1; \\ \tfrac{1}{2}\psi_{m(l)}(1-f_{cm})\,f_{\mathrm{Pr}EP} & \text{any } l; k=2;\ \mathrm{p}=2; \\ \\ \tfrac{1}{2}\psi_{m(l)}\,f_{cm}(1\text{-}f_{\mathrm{Pr}EP}) & \text{any } l; k=3;\ \mathrm{p}=1; \\ \tfrac{1}{2}\psi_{m(l)}\,f_{cm}\ f_{\mathrm{Pr}EP} & \text{any } l; k=3;\ \mathrm{p}=2; \end{cases}$$

…(3)

Force of infection

The force of infection is the per capita rate at which susceptible individuals acquire infection. The force of infection $\lambda_{l,k}^{p}$ is the force of infection experienced by individuals of each sex and circumcision status (if male), risk group and PrEP status from the infected population of the opposite sex at a given time. Characteristics of an individual ($l,\ k,$ and p) are distinguished from those of their sexual partners by means of a prime (i.e. $l',\ k'$ and p′). The force of infection depends on the pattern of partnership formation between different risk groups and on the probability of transmission per partnership, and is defined as:

for women: $$\lambda_{l,1}^{p} = \sum_{l'}\sum_{k'}\sum_{p'}\sum_{s'}\left[\rho_{g,l,l'}\left(\frac{X_{l',k'}^{s',p'}}{\sum_{k'=2}^{4}\sum_{p'}\sum_{s'}X_{l',k'}^{s',p'}}\right)z_{l,1,p,s',l',k',p'}\right]$$

for men: $$\lambda_{l,k}^{p} = \sum_{l'}\sum_{p'}\sum_{s'}\left[\rho_{g,l,l'}\left(\frac{X_{l',1}^{s',p'}}{\sum_{p'}\sum_{s'} X_{l',1}^{s',p'}}\right) Z_{l,k,p,s',l',1,p'}\right]$$

…(4)

Transmission per partnership

The probability of transmission per partnership depends on: (i) the probability of transmission per coital act; and (ii) the number of coital acts during the partnership (which depends on the risk group of each partner). The probability of transmission per coital act depends on: an individual's sex, circumcision status (if male), PrEP use and adherence (if using PrEP), as well as their partner's stage of infection (including ART use), sex, circumcision status (if male) and PrEP use, in addition to the degree of condom use in the partnership (which depends on the risk group of each partner).

A baseline transmission probability from uncircumcised males to females is assumed (β_0). The difference in acquisition and transmission per sexual act for other factors (e.g. stage of infection) is specified with respect to this baseline transmission probability using a multiplicative factor (Table S2). The probability of HIV transmission per sex act is given by $\beta_{p,k}^{s',k'}$ and depends on:

- s' (partner's HIV-status),
- k' (partner's sex, and circumcision status (if male)),
- p (individual's PrEP status) and
- k (individual's sex, and circumcision status (if male)).

Male circumcision and PrEP are assumed to reduce the risk of acquisition but not onward transmission (if breakthrough infection occurs while on PrEP). It is assumed that if men have sex during the circumcision healing period they are more likely to acquire HIV, and are also more likely to transmit HIV onwards. However, for these analyses it is also assumed that men do not have sex during the healing period.

Parameter	Value	Source
Baseline probability of transmission from an uncircumcised man to a woman, per sex act (β0)	0.0011	Calibrated value, within the range reported in a meta-analysis of per sex act heterosexual risk of HIV-1 infection [3].
Factor increase in transmission for acute HIV infection, per sex act	26	Hollingsworth et. al. [1]
Factor increase in baseline transmission for pre-AIDS stage 1 infection, per sex act	1	Hollingsworth et. al. [1]

Factor increase in baseline transmission for pre-AIDS stage 2 infection, per sex act	7	Hollingsworth et. al. [1]
Factor increase in baseline transmission for AIDS infection, per sex act	0	Hollingsworth et. al. [1]
Factor increase in baseline transmission from women to men, per sex act	0.4	Based on reduced transmission probability for female-to-male compared to male-to-female transmission [3]
Factor increase in baseline transmission from circumcised men, per sex act	1	Wawer et. al. [4]
Factor increase in acquisition of HIV from women to men, if man is circumcised, per sex act	0.4	[5-7]
Fraction increase in acquisition of HIV if HIV-negative partner is using PrEP, per sex act	0.09	Donnell et. al. [8]
Fraction increase in transmission of HIV if HIV-positive partner is on ART, per sex act	0.09	Baggaley et. al. [9]

Table S2. Factor increments in transmission probability per sex act with respect to baseline transmission probability.

The number of sex acts in a partnership depends on the risk group of both partners and is given by the matrix $n_{sex}(l,l')$. Condom use is incorporated as a proportion of sex acts in which condoms are used, via the matrix $CU(l,l')$, which defines condom use in a partnership between an individual's risk group l and their partner's risk group l'. The level of adherence to PrEP is incorporated as a proportion of sex acts protected by PrEP, for good adherers (P_1) and poor adherers (P_2).

The probability of transmission per partnership $Z_{l,k,p,s',l',k',p'}$ is defined as:

For those not receiving PrEP (p=1, p=2):

$$Z_{l,k,p,s',l',k',p'} = 1-((1-\beta_{p,k}^{s',k'})^{\bar{\chi}})$$

For those receiving PrEP and adhering well (p=3):

$$Z_{l,k,3,s',l',k',p'} = 1-((1-\beta_{3,k}^{s',k'})^{\bar{\chi}\mathrm{P}_1})((1-\beta_{3,k}^{s',k'})^{\bar{\chi}(1-\mathrm{P}_1)})$$

For those receiving PrEP and adhering poorly (p=4):

$$Z_{l,k,4,s',l',k',p'} = 1-((1-\beta_{4,k}^{s',k'})^{\bar{\chi}\mathrm{P}_2})((1-\beta_{4,k}^{s',k'})^{\bar{\chi}(1-\mathrm{P}_2)})$$

…(5)

where:

$$\overline{\chi} = (1 - CU(l,l'))n_{sex}(l,l')$$

...(6)

That is, $\overline{\chi}$ is the number of sex acts not protected by condoms in a partnership between an individual of risk group l and their partner of risk group l'.

Sexual mixing

The mixing pattern is defined with respect to sex and behavioural risk group. The number of sexual partnerships that an individual of sex g (where g=1 refers to females and g=2 to males), and behavioural risk group l forms with an individual of the opposite sex, and risk group l', is given by $\rho_{g,l,l'}$, and is defined as:

for females:

$$\rho_{1,l,l'} = c_{1,l}\left(\varepsilon\delta_{l,l'} + (1-\varepsilon)\left(\frac{c_{2,l'}\sum_{k'=2}^{4}\sum_{p'}\sum_{s'}X_{l',k'}^{s',p'}}{\sum_{l'=1}^{3}c_{2,l'}\sum_{k'=2}^{4}\sum_{p'}\sum_{s'}X_{l',k'}^{s',p'}}\right)\right)$$

for males:

$$\rho_{2,l,l'} = c_{2,l}\left(\varepsilon\delta_{l,l'} + (1-\varepsilon)\left(\frac{c_{1,l'}\sum_{p'}\sum_{s'}X_{l',1}^{s',p'}}{\sum_{l'=1}^{3}c_{1,l'}\sum_{p'}\sum_{s'}X_{l',1}^{s',p'}}\right)\right)$$

...(7)

The parameter $c_{g,l}$ gives the mean number of partners in a year per individual of sex g and risk group l. The degree of assortativity in mixing with respect risk is given by ε , whereby a value of 1 indicates fully assortative mixing. The identity matrix with respect to risk is given by the Kronecker delta $\delta_{l,l'}$, whereby:

$$\delta_{l,l'} = \begin{cases} 1, & if\ l = l' \\ 0, & if\ l \neq l' \end{cases}$$

In order to balance the number of sexual partnerships, a discrepancy matrix D_{l_2,l_1} is defined to calculate the discrepancy between males and females regarding the number of partnerships formed with respect to each risk group [10], where l_2 is the risk group of the male partner, and l_1 is the risk group of the female partner. D_{l_2,l_1} is calculated as:

$$D_{l_2,l_1} = \frac{\rho_{2,l,l'} \sum_{k=2}^{4} \sum_{p} \sum_{s} X_{l,k}^{s,p}}{\rho_{1,l,l'} \sum_{p} \sum_{s} X_{l,1}^{s,p}}$$

…(8)

The extent to which balancing of the number of sexual partnerships is male-driven or female-driven is determined by the parameter θ. When $\theta = 1$, balancing is male-driven, that is, the number of partnerships formed by females is adjusted such that the number of partnerships formed by females is equal to the number formed by males. Equally, when $\theta = 0$, balancing is female-driven, and when $\theta = 0.5$ the sexes compromise equally. Balancing of sexual partnerships is carried out with respect to both partner's risk groups and is represented by:

$$\rho_{2,l,l'} \rightarrow D_{l_2,l_1}{}^{(\theta-1)} \rho_{2,l,l'}$$

$$\rho_{1,l,l'} \rightarrow D_{l_2,l_1}{}^{(\theta)} \rho_{1,l,l'}$$

…(9)

Time-limited transmission between migrant miners and their partners in Gaza

All transmission between the miners and their partners in Gaza is assumed to happen in December each year, whereby for the first 11 months of each year:

- $\rho_{1,3,3}(t) = 0$
- $\rho_{2,3,3}(t) = 0$

The miners experience an additional force of infection ϑ for the first 11 months of each year (i.e. from their partners in South Africa), whereby for the first 11 months of each year:

- $\lambda_{3,k}^{p}(t) = \lambda_{3,k}^{p} + \vartheta$

Parameter	Symbol	Value	Source
Mixing parameter<br>(the extent to which sexual mixing is assortative)	ε	0.9	Calibrated
Balancing parameter<br>(the extent to which balancing the number of sexual partnerships is male or female-driven)	θ	0.5	Assumed
Force of infection experienced in South Africa by the migrant miners for the first 11 months of each year	ϑ	2.7 per 100 PY	Calibrated

Table S3. Parameters regarding sexual mixing and transmission which occurs at the mines in South Africa.

Male circumcision intervention

To simulate the intervention moving uncircumcised men to being circumcised, the function $g(k)$ is included in equations (1):

$$for \;\; t \le t_{Circ}$$

$$\eta_C = 0$$

$$for \;\; t > t_{Circ}$$

$$\begin{aligned} g(1) &= 0 \\ g(2) &= -\eta_C X_{l,2}^{s,p} \\ g(3) &= +\eta_H X_{l,4}^{s,p} \\ g(4) &= +\eta_C X_{l,2}^{s,p} - \eta_H X_{l,4}^{s,p} \end{aligned}$$

…(10)

The parameter η_C gives the scale-up rate for male circumcision and η_H gives the rate of wound healing following circumcision (assumed to be a mean duration of 6 weeks). It is assumed that men can be circumcised regardless of infection status, and if infected, regardless of stage of infection. The scale-up of male circumcision assumed in the model is shown in Figure S2.

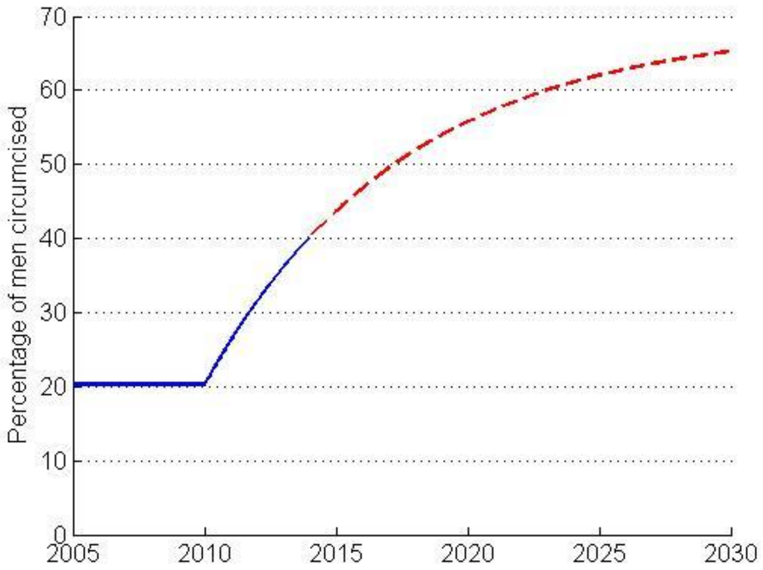

Figure S2: The prevalence of male circumcision over time assumed in the model.

Antiretroviral Therapy

In the model, ART initiation is possible from each stage of infection ($\phi_1 \ldots \phi_5$). Individuals initiating ART with CD4 >350 cells or CD4 <350 cells are moved to $X_{l,k}^{7,p}$ or $X_{l,k}^{8,p}$ respectively and are assumed to survive on average $1/\omega_E$ or $1/\omega_L$ years before progressing to the AIDS stage. Individuals drop out from early and late ART at a rate of ξ_E and ξ_L, respectively, both assumed to be 5 per 100 PY. The number of individuals assumed to be receiving ART with respect to time is shown in Figure S3.

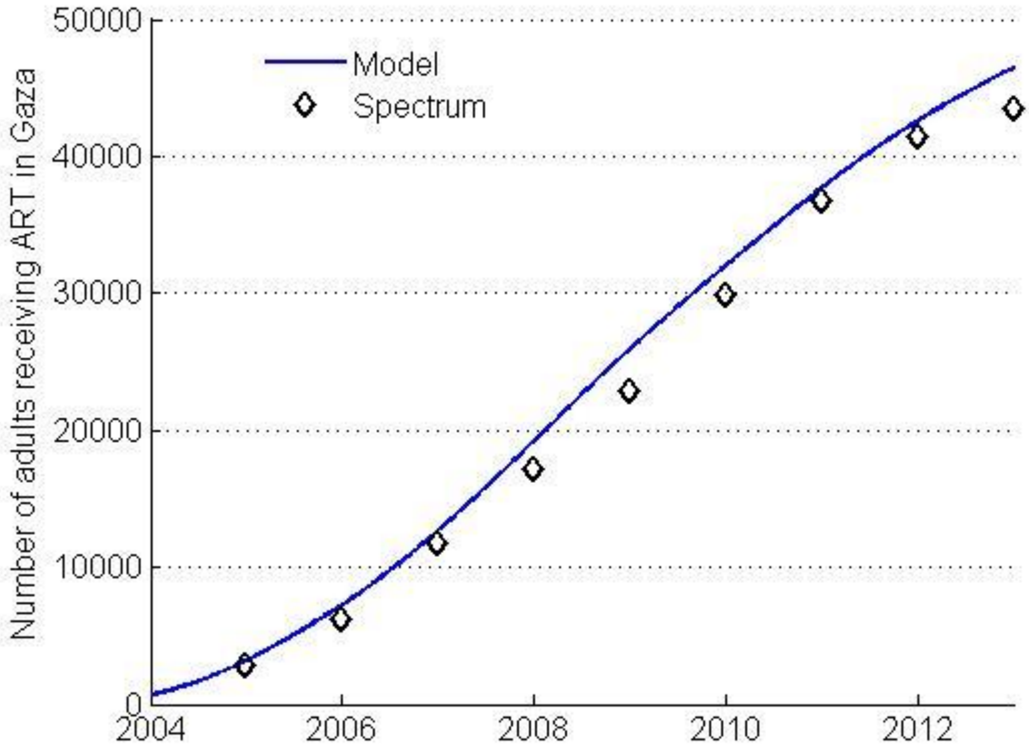

Figure S3: The number of adults receiving ART in Gaza assumed in the model compared to data reported to UNAIDS (Spectrum (v4.7)).

PrEP intervention

To simulate an intervention whereby PrEP is provided to uninfected individuals, the function $h(l, p)$ is included in the equation for susceptible individuals (1):

$$
\begin{aligned}
h(l,1) &= 0 \\
h(l,2) &= -\eta_{P(l)} X_{l,k}^{0,2} \\
h(l,3) &= + f_a \eta_{P(l)} X_{l,k}^{0,2} \\
h(l,4) &= +(1 - f_a) \eta_{P(l)} X_{l,k}^{0,2}
\end{aligned}
$$

…(11)

The parameter f_a represents the proportion of PrEP users that are 'good' adherers and the parameter $\eta_{P(l)}$ represents PrEP initiation, such that:

$for \quad t \leq t_{\Pr EP}$

$$\eta_{P(l)} = 0$$

$for \quad t > t_{\Pr EP}$

$$for \ women: \qquad \eta_{P(l)} = P_l - \left[\frac{\sum_{p=3}^{4} X_{l,1}^{0,p}}{\sum_{p=1}^{4} X_{l,1}^{0,p}} \right]$$

...(12)

where $t_{\Pr EP}$ is the time at which implementing the PrEP intervention begins and P_l is PrEP coverage for a given risk group. When modelling time-limited PrEP for Partners of miners (l=3), $\eta_{P(3)}$ is zero until week 46. At week 46 $\eta_{P(3)}$ is calculated as above, to represent PrEP initiation in this group. At week 52 this group stops using PrEP:

$$h(3,1) = 0$$
$$h(3,2) = X_{3,1}^{0,3} + X_{3,1}^{0,4}$$
$$h(3,3) = -X_{3,1}^{0,3}$$
$$h(3,4) = -X_{3,1}^{0,4}$$

...(13)

References

1. Hollingsworth TD, Anderson RM, Fraser C. HIV-1 transmission, by stage of infection. *J Infect Dis* 2008,**198**:687-693.
2. Wandel S, Egger M, Rangsin R, Nelson KE, Costello C, Lewden C*, et al.* Duration from seroconversion to eligibility for antiretroviral therapy and from ART eligibility to death in adult HIV-infected patients from low and middle-income countries: collaborative analysis of prospective studies. *Sex Transm Infect* 2008,**84 Suppl 1**:i31-i36.
3. Boily MC, Baggaley RF, Wang L, Masse B, White RG, Hayes RJ*, et al.* Heterosexual risk of HIV-1 infection per sexual act: systematic review and meta-analysis of observational studies. *Lancet Infect Dis* 2009,**9**:118-129.
4. Wawer MJ, Makumbi F, Kigozi G, Serwadda D, Watya S, Nalugoda F*, et al.* Circumcision in HIV-infected men and its effect on HIV transmission to female partners in Rakai, Uganda: a randomised controlled trial. *Lancet* 2009,**374**:229-237.
5. Auvert B, Taljaard D, Lagarde E, Sobngwi-Tambekou J, Sitta R, Puren A. Randomized, controlled intervention trial of male circumcision for reduction of HIV infection risk: the ANRS 1265 Trial. *PLoS Med* 2005,**2**:e298.
6. Bailey RC, Moses S, Parker CB, Agot K, Maclean I, Krieger JN*, et al.* Male circumcision for HIV prevention in young men in Kisumu, Kenya: a randomised controlled trial. *Lancet* 2007,**369**:643-656.
7. Gray RH, Kigozi G, Serwadda D, Makumbi F, Watya S, Nalugoda F*, et al.* Male circumcision for HIV prevention in men in Rakai, Uganda: a randomised trial. *Lancet* 2007,**369**:657-666.
8. Donnell D, Baeten JM, Bumpus NN, Brantley J, Bangsberg DR, Haberer JE*, et al.* HIV Protective Efficacy and Correlates of Tenofovir Blood Concentrations in a Clinical Trial of PrEP for HIV Prevention. *J Acquir Immune Defic Syndr* 2014,**66**:340-348.
9. Baggaley RF, White RG, Hollingsworth TD, Boily MC. Heterosexual HIV-1 infectiousness and antiretroviral use: systematic review of prospective studies of discordant couples. *Epidemiology* 2013,**24**:110-121.
10. Garnett GP, Anderson RM. Balancing sexual partnerships in an age and activity stratified model of HIV transmission in heterosexual populations. *IMA J Math Appl Med Biol* 1994,**11**:161-192.
